# Vascular Stem and Progenitor Cells in Diabetic Complications

**DOI:** 10.1155/2012/580343

**Published:** 2012-05-15

**Authors:** Gian Paolo Fadini, Paolo Madeddu, Johannes Waltenberger, Paolo Fiorina

**Affiliations:** ^1^Department of Medicine, University of Padua, Via Giustiniani 2, 35100 Padua, Italy; ^2^Laboratory of Experimental Diabetology, Venetian Institute of Molecular Medicine, 35129 Padua, Italy; ^3^Bristol Heart Institute, Regenerative Medicine Section, School of Clinical Sciences, University of Bristol/Bristol Royal Infirmary, Level 7, Bristol BS2 8HW, UK; ^4^Department of Cardiology and Angiology, University Hospital Münster, Albert-Schweitzer Campus 1-A1, 48149 Münster, Germany; ^5^Transplantation Research Center, Division of Nephrology, Children's Hospital Boston, 300 Longwood Avenue, Boston, MA 02115, USA; ^6^Department of Medicine, San Raffaele Scientific Institute, 20132 Milan, Italy

Hyperglycemia and its associated biochemical abnormalities damage vascular wall cells, especially the endothelium, leading to an increased risk of cardiovascular events and disease, as well as microangiopathy and end-organ complications. In the last decade, accumulating data suggest that vascular repair mechanisms are important to maintain normal homeostasis of the arterial wall and to prevent development of pathologic processes, such as atherosclerosis, restenosis, and microvascular disease. 

Diabetes mellitus, through the impairment of vascular stem and progenitor cells, entails a defective repair of the injured endothelium. The biochemical and cellular mechanisms that account for reduced or functionally impaired vascular progenitor cells in diabetes are not fully elucidated, and this is an intense area of research. Additionally, therapeutic approaches to modulate the endogenous reparative/regenerative processes are of particular interest in the setting of experimental and clinical diabetes research.

For this special issue of Experimental Diabetes Research, we invited investigators to contribute with original research articles and review articles that stimulate the continuing efforts to understand the molecular and cellular aspects underlying defective vascular repair by means of stem/progenitor cells in diabetes, as well as the development of interventions to reverse it. 

The journal has received a variety of valuable submissions spanning the pathophysiological and therapeutic implications of vascular stem/progenitor cells.

The pathophysiological implications are herein described in the setting of both diabetes and the metabolic syndrome. S. Devaraj and I. Jialal report how number and/or functionality of endothelial progenitor cells (EPCs) could emerge as a novel cellular biomarker of endothelial/vascular dysfunction and cardiovascular disease (CVD) risk in patients with the metabolic syndrome. In the setting of diabetes, a focus review highlights the central contribution played by bone-marrow-derived progenitor cells in the development and progression of chronic complications. Not only are EPCs reduced and dysfunctional in diabetes, but they also appear to have a deranged differentiation capacity, which is shifted toward a procalcific phenotype that may have a negative impact on ectopic calcification and atherosclerosis. Of note, circulating progenitor cell phenotypes are not limited to EPC, but may include a variety of lineage-committed cells relevant for the pathobiology of diabetic complications. As an example, the level of pericyte progenitor cells (PPCs) in type 2 diabetes appears to be related to microangiopathy in response to glucose-lowering therapy. Among disparate complications, retinopathy has received a special attention: while G. Tremolada and colleagues provide a comprehensive analysis of the mechanisms of neoangiogenesis in the diabetic retina, R. Longeras et al. show how pigment-epithelium-derived-factor- (PEDF-) 34 attenuates EPC mobilization from the bone marrow into the bloodstream during retinal neovascularization. This therapeutic approach can now be considered part of the armamentarium available to reverse microangiopathy, through regenerative cells. In parallel, S. Bernardi et al. provided an analysis of cell-based strategies to counter diabetic complications that have been so far devised and applied in the experimental and clinical settings. Besides cell therapies, several other pharmacologic and nonpharmacologic approaches have shown ability to reverse EPCs dysfunction in diabetes.

In conclusion, this special issue provides a series of updated reviews on vascular stem/progenitor cell defects in diabetes and on the therapeutic approaches to reverse them and counter diabetic complications. Original contributions help us to dissect the complexity of vascular stem/progenitor cell biology and trace the way for future studies in this field. 

Amazingly, circulating progenitor cells are uncovering an entirely new scenario in diabetology research: it is all in the blood!

*Gian Paolo Fadini* *Gian Paolo Fadini* *Paolo Madeddu* *Paolo Madeddu* *Johannes Waltenberger* *Johannes Waltenberger* *Paolo Fiorina* *Paolo Fiorina*

